# Supplementary material for: Structural basis of complex formation between mitochondrial anion channel VDAC1 and Hexokinase-II
Source: Commun Biol. 2021 Jun 3;4:667. doi: 10.1038/s42003-021-02205-y (PMC8175357; doi:10.1038/s42003-021-02205-y)
Supplement: Supplementary file 1 — Supplementary Information [file 42003_2021_2205_MOESM1_ESM.pdf]

Structural Basis of Complex Formation Between Mitochondrial Anion Channel VDAC1 and Hexokinase-II (Supporting Information)

Nandan Haloi[1,2,3], Po-Chao Wen[1,2,3], Qunli Cheng[4], Meiying Yang[4], Gayathri Natarajan[4], Amadou KS Camara[4,5,6], Wai-Meng Kwok[4,6,7,*], and Emad Tajkhorshid[1,2,3,*]

[1]Theoretical and Computational Biophysics Group, NIH Center for Macromolecular Modeling and Bioinformatics, Beckman Institute for Advanced Science and Technology, University of Illinois at Urbana-Champaign, Urbana, IL 61801
[2]Department of Biochemistry, University of Illinois at Urbana-Champaign, Urbana, IL 61801
[3]Center for Biophysics and Quantitative Biology, University of Illinois at Urbana-Champaign, Urbana, IL 61801
[4]Department of Anesthesiology, Medical College of Wisconsin, Milwaukee, WI 53226
[5]Department of Physiology, Medical College of Wisconsin, Milwaukee, WI 53226
[6]Cancer Center and Cardiovascular Center, Medical College of Wisconsin, Milwaukee, WI 53226
[7]Department of Pharmacology & Toxicology, Medical College of Wisconsin, Milwaukee, WI 53226
[*]Correspondence: wmkwok@mcw.edu or emad@illinois.edu

Table S1. Population and BD replica contributions for the five clusters.

Cluster	Population (%)	No. of BD replicas contributing to each cluster
1	14.0	25
2	9.3	13
3	6.0	10
4	4.8	7
5	4.6	10

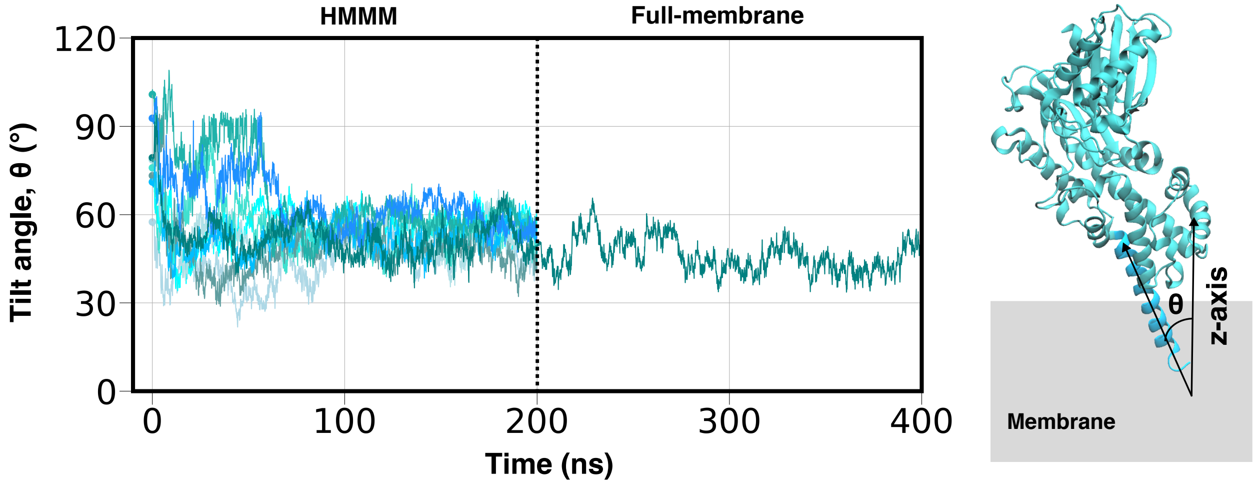

Figure S1. Tilt angle of the H-anchor relative to the membrane normal (*z*-axis). The vertical dashed line separates the initial, HMMM membrane-binding simulation (left side, all 9 successful replicas shown), from the following 200-ns, full-membrane simulation performed for one of the systems (right side).

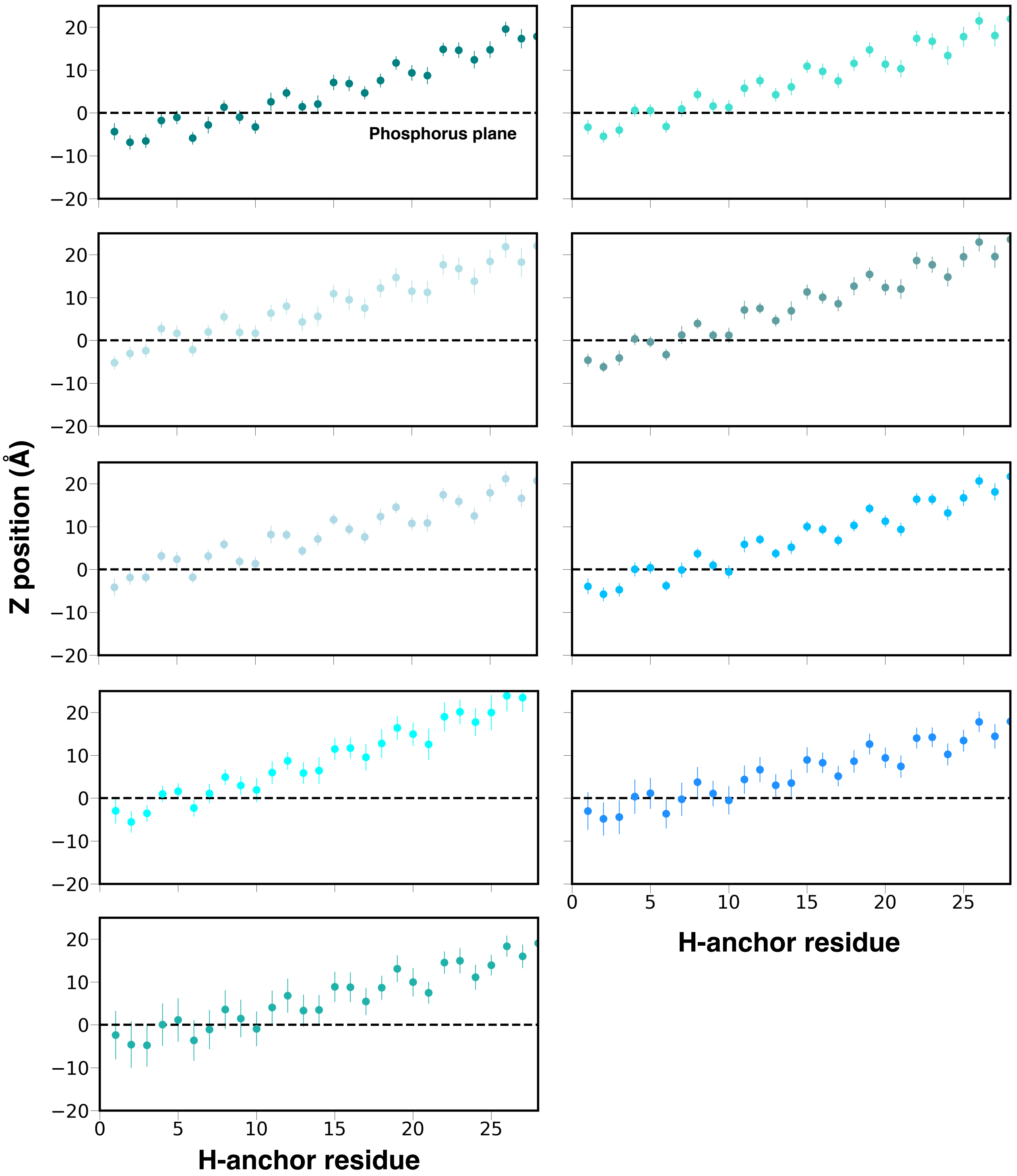

Figure S2. Mean and standard deviation of the *z* positions (relative to the cis phosphorus plane) of the center of mass (C.O.M) of the H-anchor residues (for all 9 successful replicas), calculated from the last 150 ns of each membrane-bound HMMM simulations.

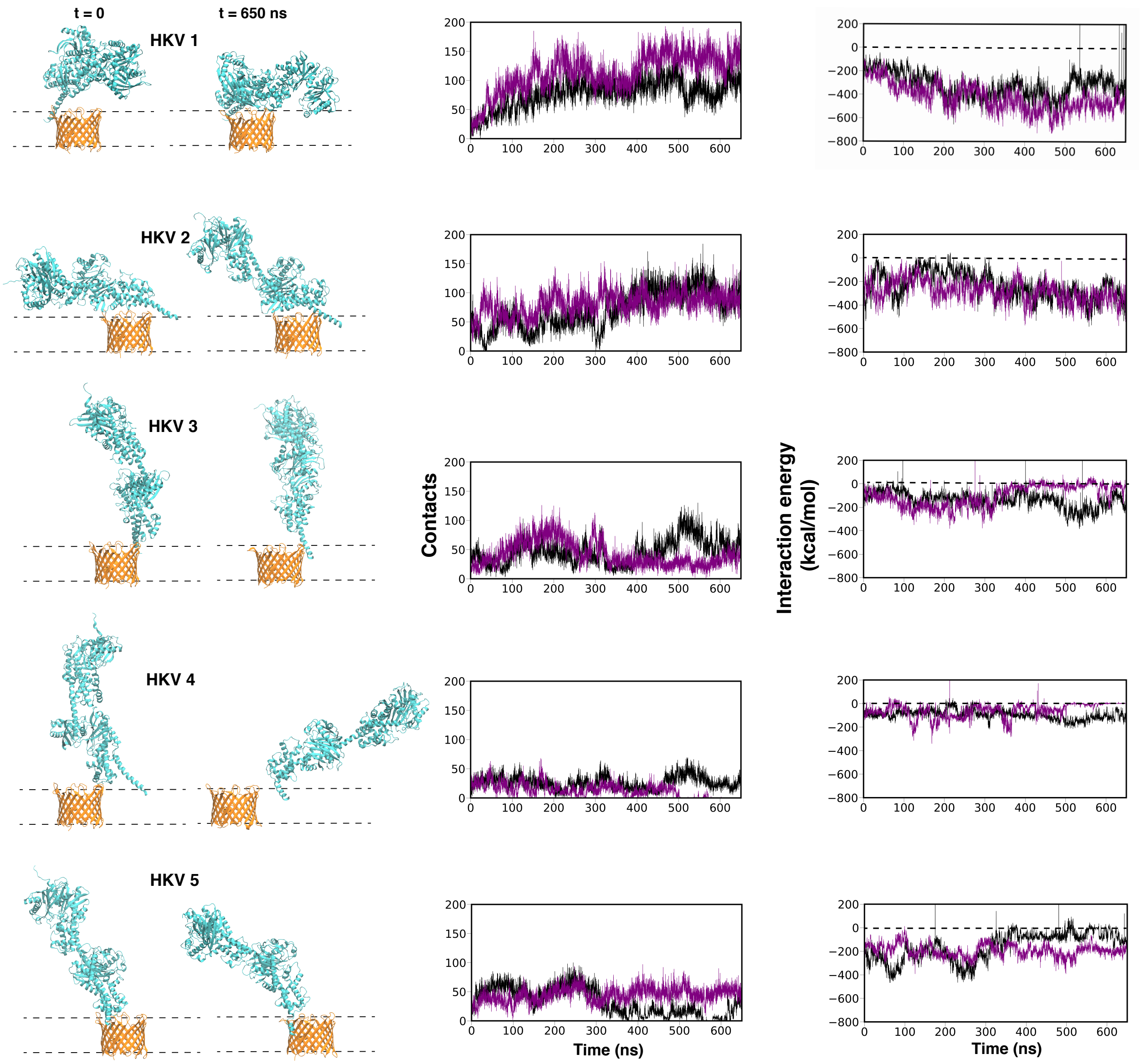

Figure S3. (*Left*) Molecular representation of membrane-embedded HKV1, HKV2, HKV3, HKV4 and HKV5. Relative orientations of VDAC1 is kept similar in all the complexes for better comparison. (*Right*) Time evolution of the number of contacts and interaction energy (van der Waals + electrostatic) between HKII and VDAC1 during MD simulation were plotted for each complex (each replica colored differently).

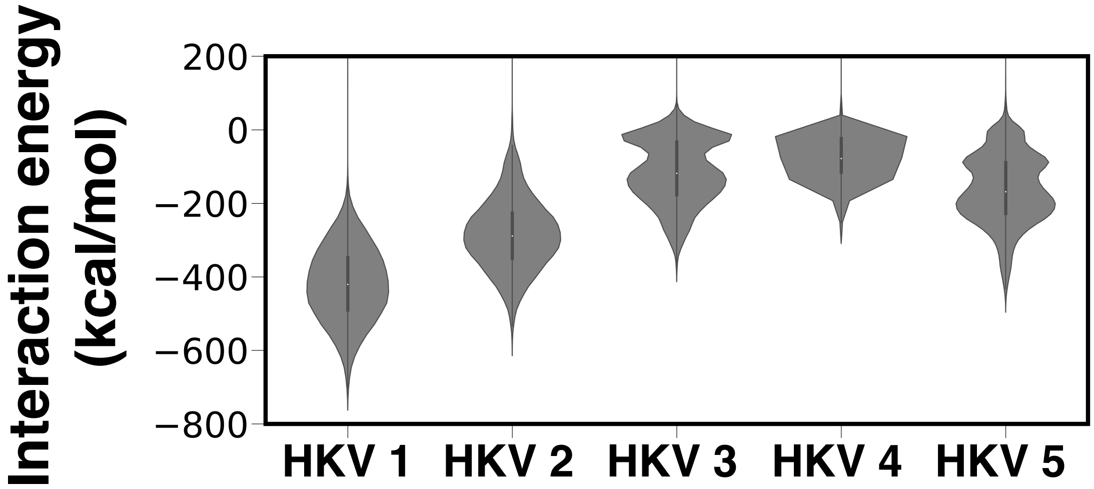

Figure S4. Violin plots of HKII/VDAC1 interaction energy for all the five complexes, derived from the last 450 ns of MD simulations of both replicas for each complex.

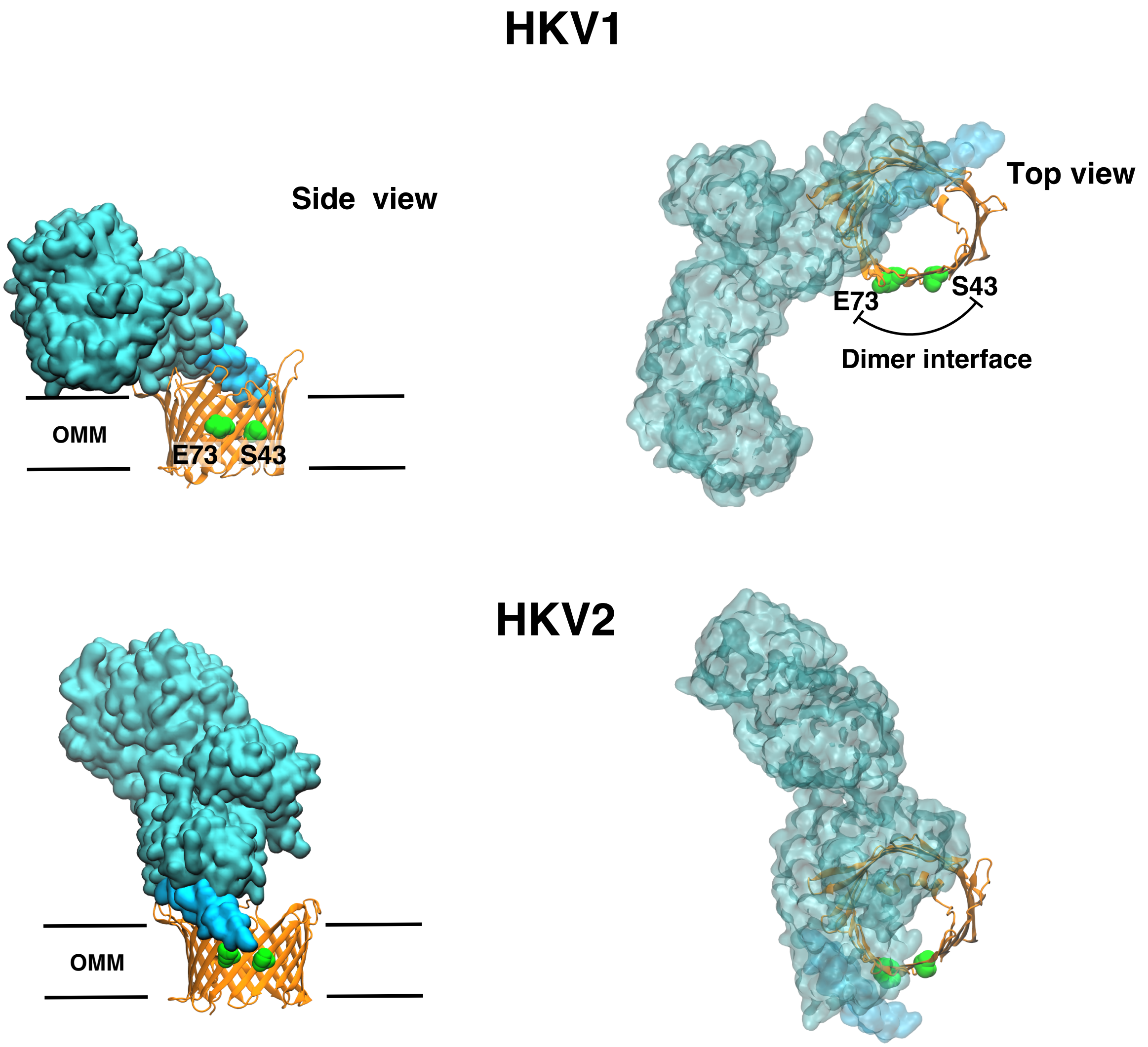

Figure S5. Positioning of HKII conflicts with a putative dimeric interface, formed by the β-barrel face composed of E73 and S43[1], in HKV2, but not in HKV1.

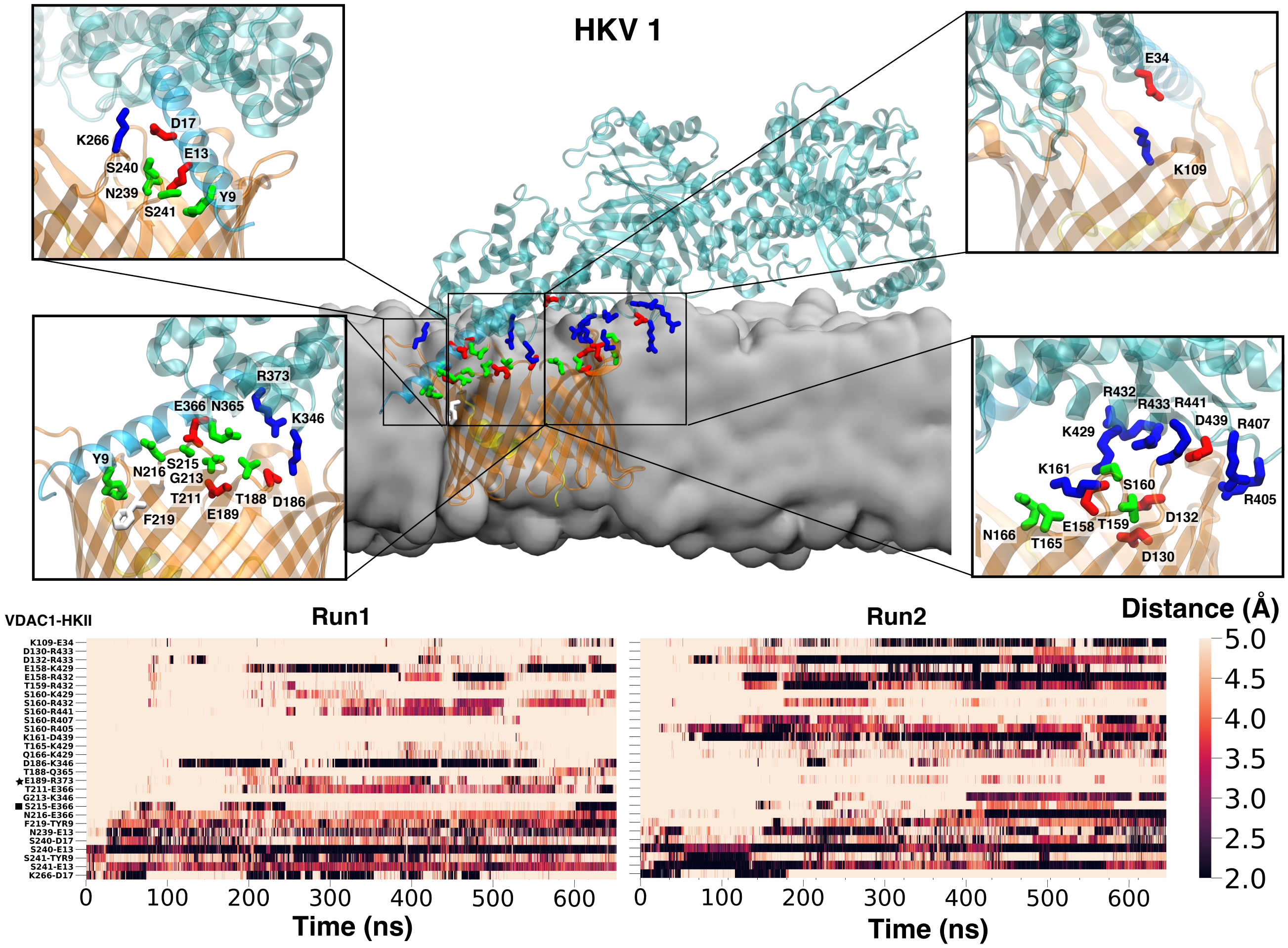

Figure S6. Residue-level analysis of VDAC1-HKII interactions in HKV1. (*Top*) Hydrogen bond and salt-bridge interactions between VDAC1 and HKII maintaining the structure of HKV1. Interactions with >30% probability during the MD simulation (in the last 450 ns) of either replica are highlighted in the *insets*. Interaction probabilities are calculated using a distance cutoff of 5 Åbetween any atoms of the two proteins. The membrane is shown in gray. Each protein residue is colored based on its type: gray representing hydrophobic, green polar, red acidic, and blue basic residues. (*Bottom*) Heat map of distances of multiple salt-bridge and hydrogen-bond pairs. A star indicates an interaction pair involving VDAC1:E189, which has been identified to be important for HKI interaction[2]. A square indicates an interaction pair involving VDAC1:S215.

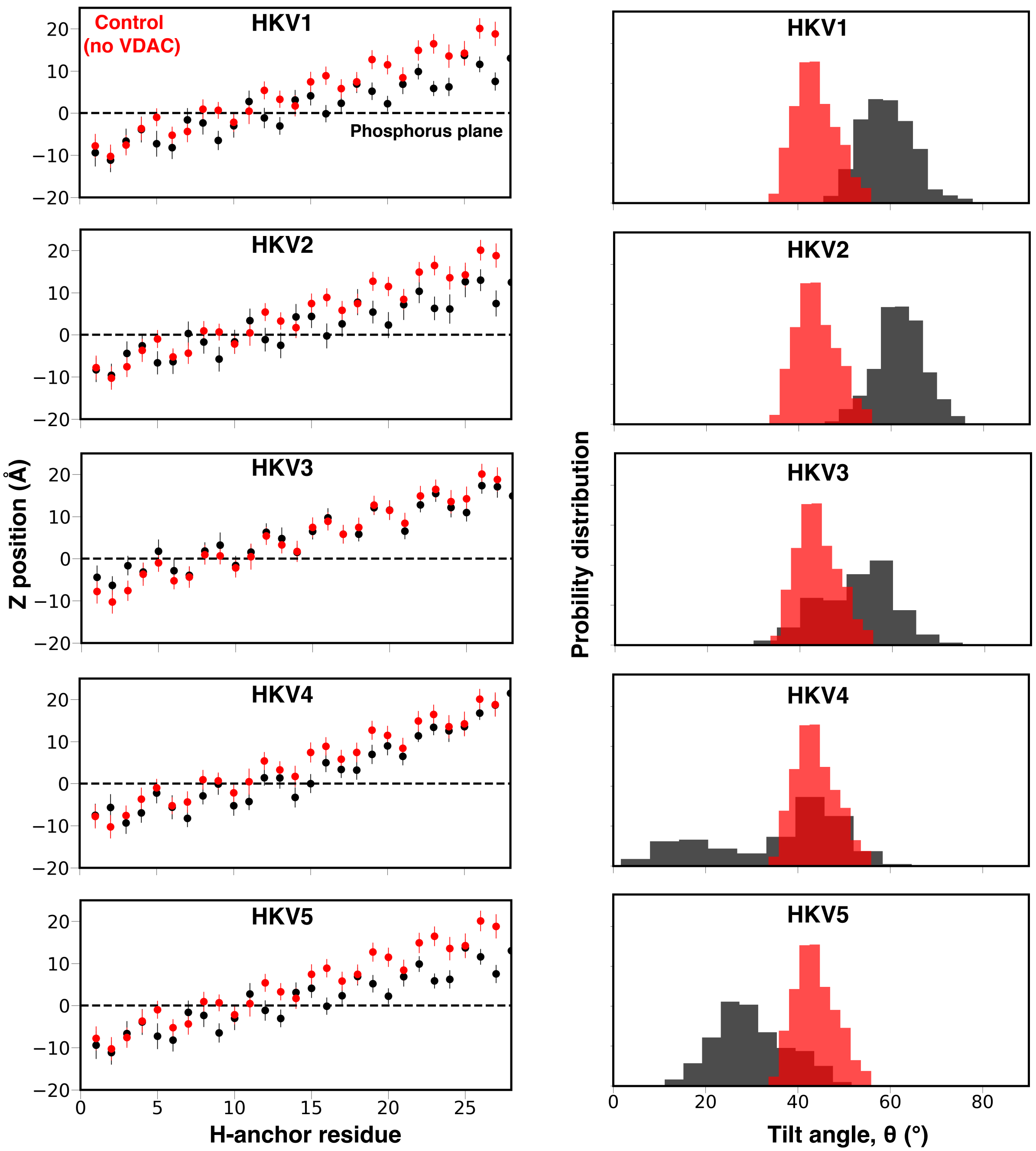

Figure S7. Membrane insertion depth and tilt angle distribution of H-anchor in each HKII/VDAC1 complex. (*Left*) Mean and standard deviation of the *z* positions (relative to the cis phosphorus plane) of the C.O.M of the H-anchor residues in all HKII/VDAC1 complexes (black), averaged over the last 450 ns of MD simulation of two replicas. For comparison, membrane partitioning of the H-anchor alone obtained from the full-membrane MD simulations (without VDAC) is also shown (red). (*Right*) Tilt angle (relative to the membrane normal) distribution of H-anchor in all HKII/VDAC1 complexes (black). For comparison, the tilt angle distribution obtained from the full-membrane MD simulation (without VDAC) is also shown (red).

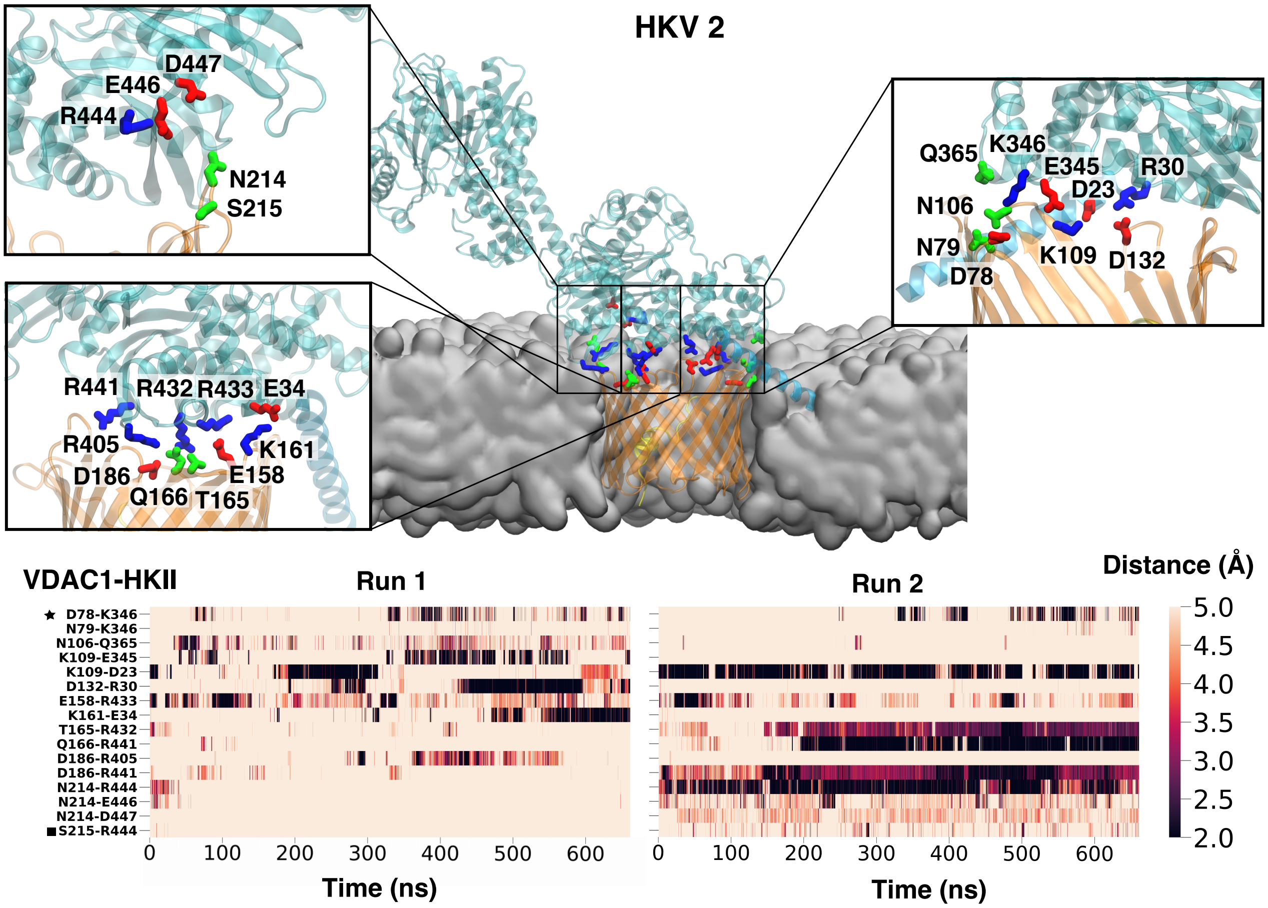

Figure S8. Residue-level analysis of VDAC1-HKII interactions in HKV2. (*Top*) Hydrogen bond and salt-bridge interactions between VDAC1 and HKII in HKV2. Interactions with >30% probability during the MD simulation (in the last 450 ns) of either replica are shown in the *insets*. Probabilities are calculated using a distance cutoff of 5 Åbetween any atoms of the two proteins. The coloring scheme is the same as in Fig. S6. (*Bottom*) Heat map of distances of multiple salt-bridge and hydrogen-bond pairs. A star indicates an interaction pair involving VDAC1:D78, which has been previously identified to be important for HKI interaction[2]. A square indicates an interaction involving VDAC1:S215.

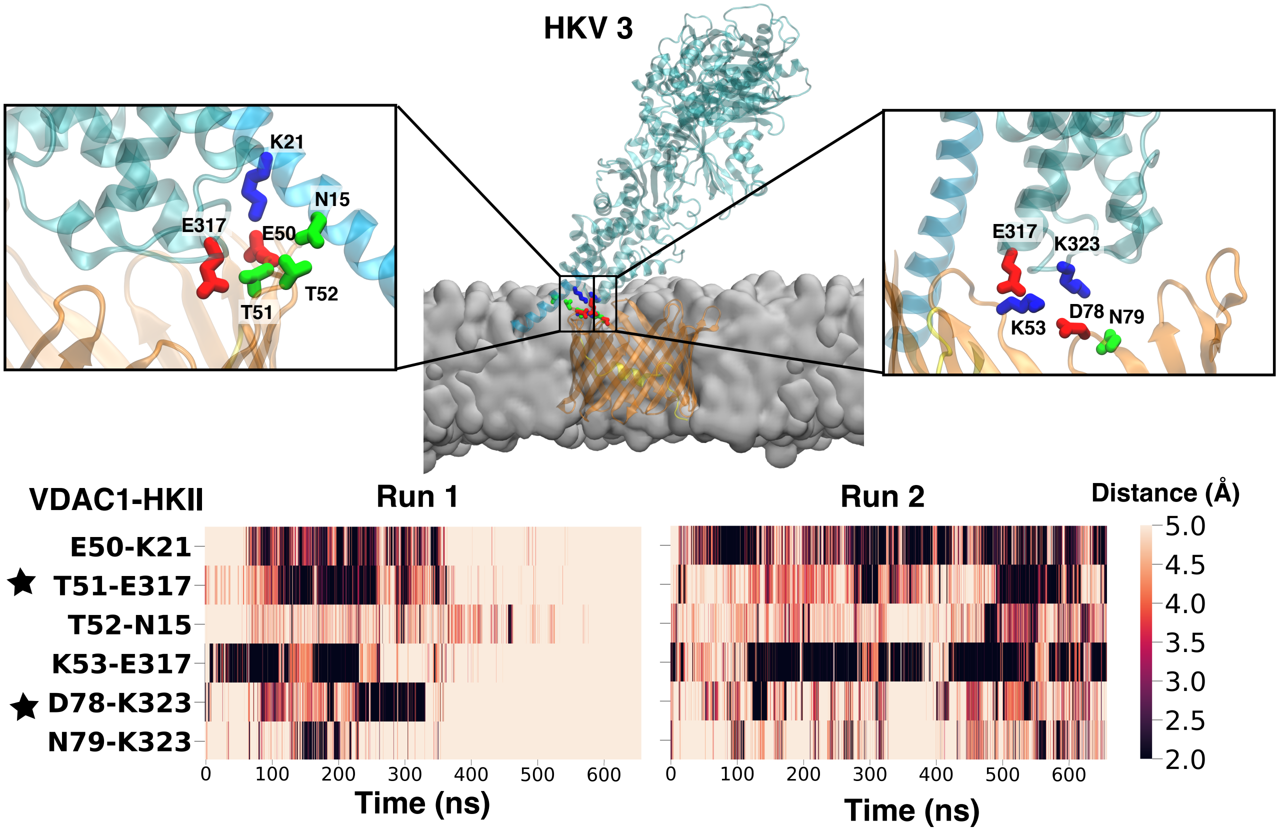

Figure S9. Residue-level analysis of VDAC1-HKII interactions in HKV3. (*Top*) Hydrogen bond and salt-bridge interactions between VDAC1 and HKII in HKV3. Interactions with >30% probability during the MD simulation (in the last 450 ns) of either replica are shown in the *insets*. Probabilities are calculated using a distance cutoff of 5 Åbetween any atoms of the two proteins. The coloring scheme is the same as in Fig. S6. (*Bottom*) Heat map of distances of multiple salt-bridge and hydrogen-bond pairs. A star indicates an interaction pair involving VDAC1:D78 or VDAC1:T51, both reported previously to be important for HKII (or HKI) interaction[2,3]. All interactions were lost after 500 ns in one replica, suggesting the instability of the HKV3 complex.

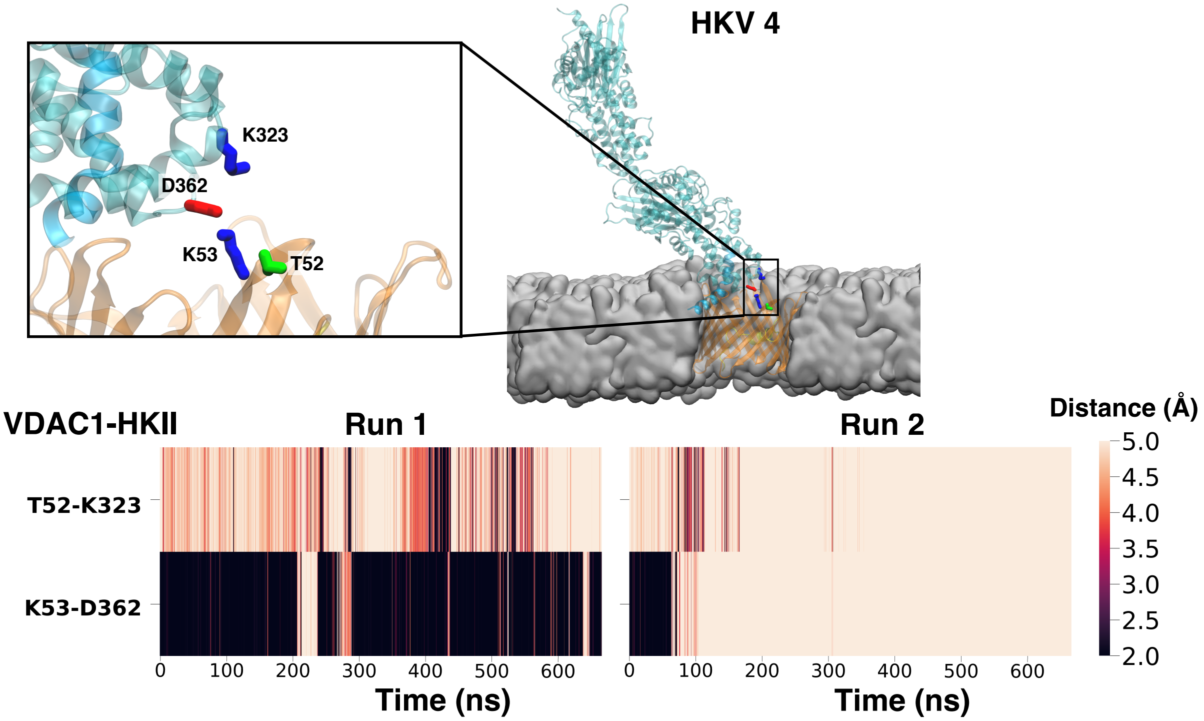

Figure S10. Residue-level analysis of VDAC1-HKII interactions in HKV4. (*Top*) Hydrogen bond and salt-bridge interactions between VDAC1 and HKII in HKV4. Interactions with >30% probability during the MD simulation (in the last 450 ns) of either replica are shown in the *inset*. Probabilities are calculated using a distance cutoff of 5 Å between any atoms of the two proteins. Coloring is the same as in Fig. S6. (*Bottom*) Heat map of distances of multiple salt-bridge and hydrogen-bond pairs. All interactions were lost after 200 ns in one replica, suggesting the instability of HKV4.

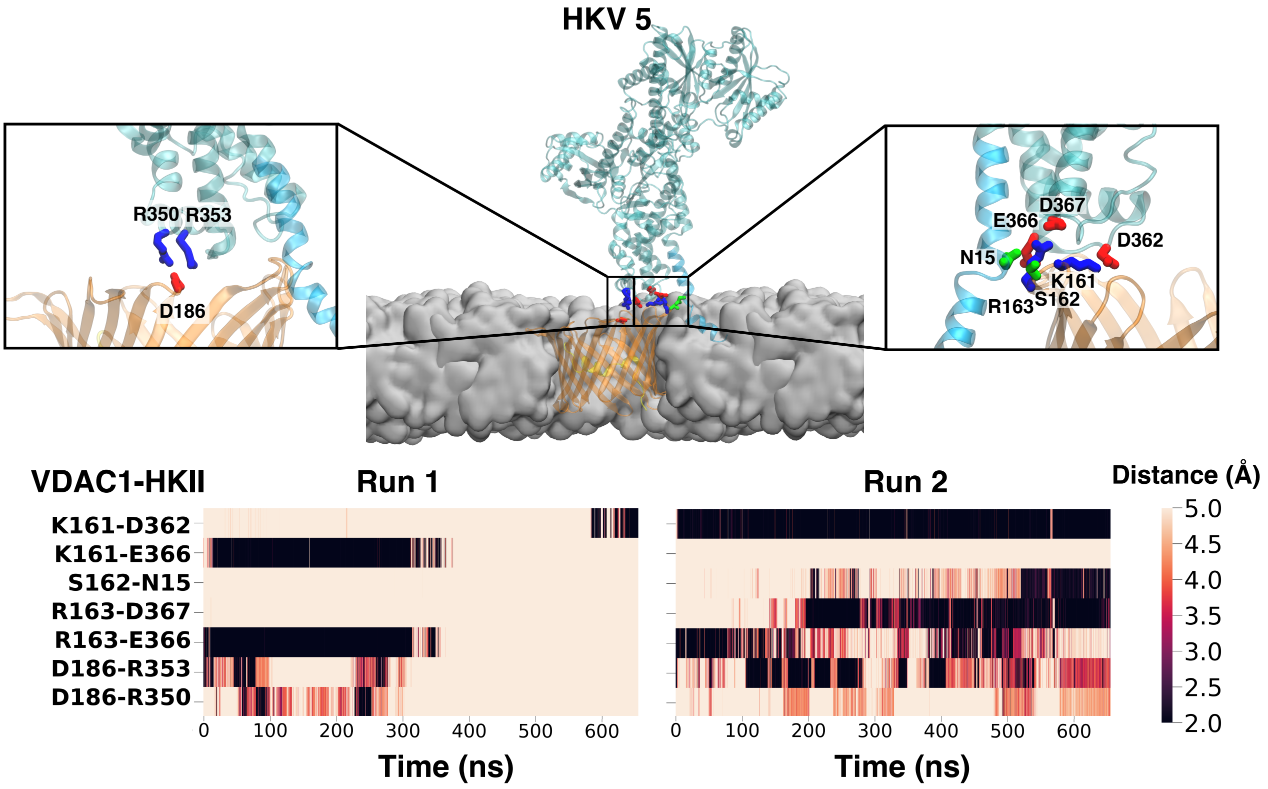

Figure S11. Residue-level analysis of VDAC1-HKII interactions in HKV5. (*Top*) Hydrogen bond and salt-bridge interactions between VDAC1 and HKII in HKV5. Interactions with >30% probability during the MD simulation (in the last 450 ns) of either replica are shown in the *insets*. Probabilities are calculated using a distance cutoff of 5 Åbetween any atoms of the two proteins. Coloring is the same as in Fig. S6. (*Bottom*) Heat map of distances of multiple salt-bridge and hydrogen-bond pairs. Nearly all interactions were lost after 400 ns in one replica, suggesting the instability of HKV5.

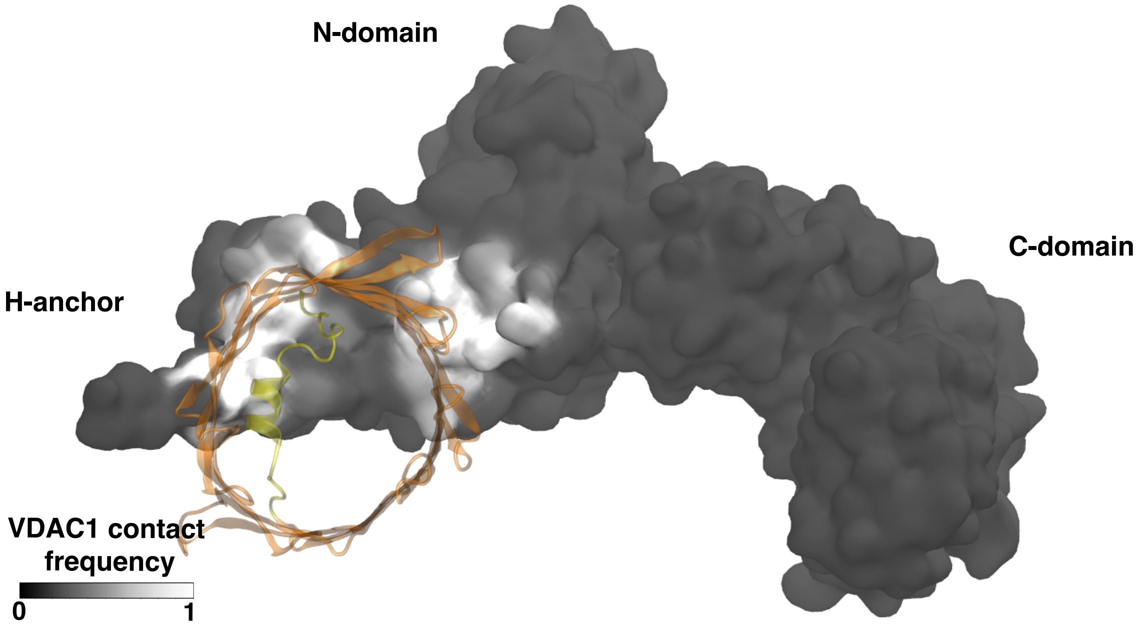

Figure S12. VDAC1 contact frequencies mapped onto the surface of HKII (black and white), viewed from the mitochondrial side and perpendicular to the membrane surface. The calculation was performed using the last 450 ns of MD simulation of both replicas. VDAC1 is shown in transparent representation, colored in orange.

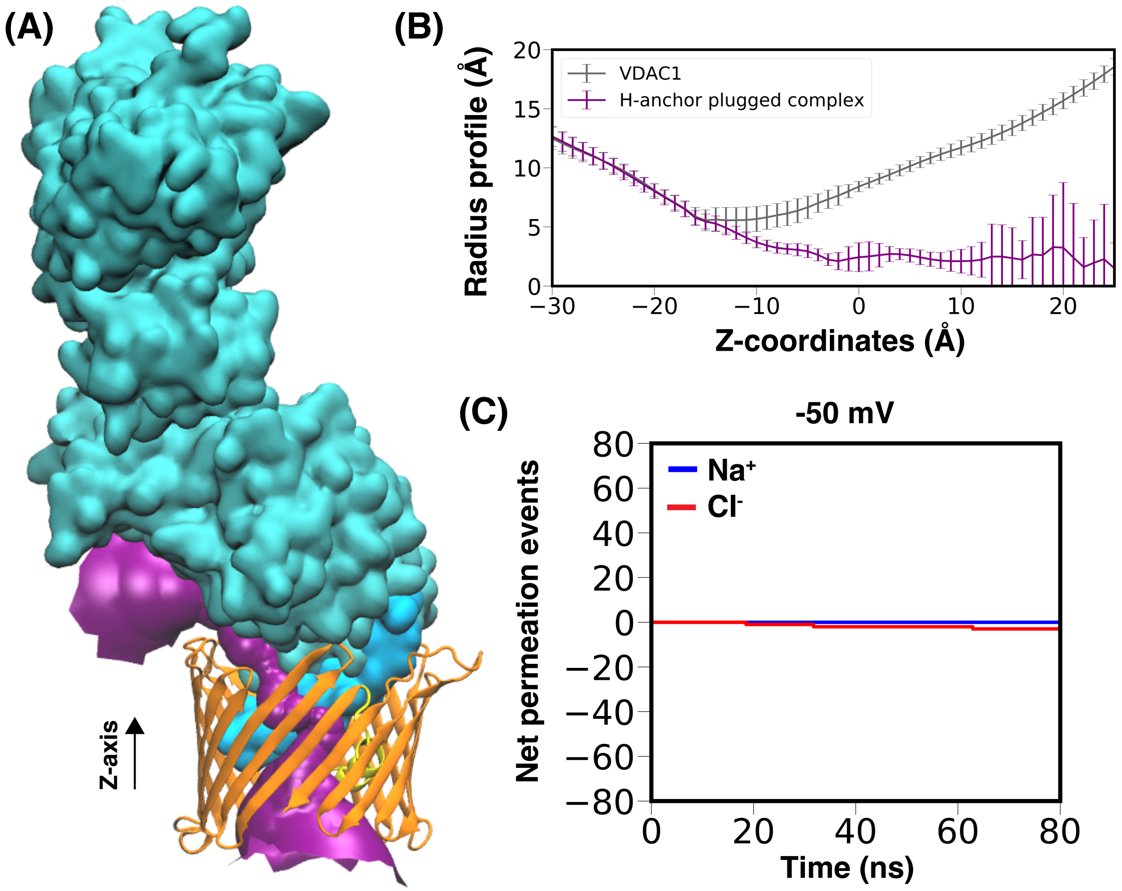

Figure S13. HKII/VDAC1 interaction in a H-anchor plugged model. (A) Purple surface showing VDAC1 pore radius profile in HKV1, made with the program HOLE[4]. Z-axis represents the membrane normal. (B) The average radius of the VDAC1 pore calculated using HOLE[4]. (C) The cumulative net number of channel-crossing events by Cl^- (red traces) and Na^+ (blue traces), tracked over the time course of the electric field MD simulation at −50 mV for the H-anchor plugged complex.

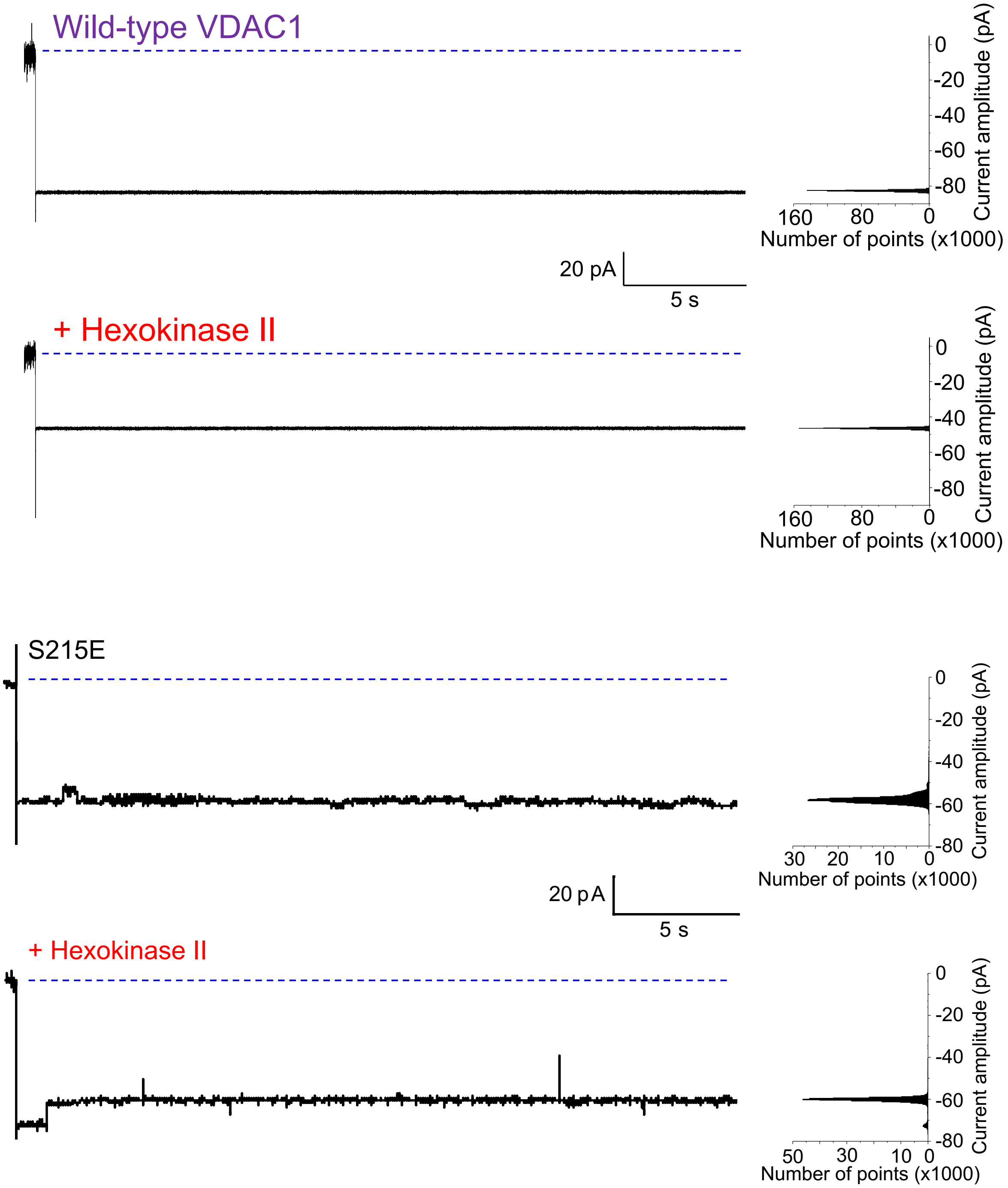

Figure S14. Effect of HKII on the conduction of wt-VDAC1 (top panel) and S215E phosphomimetic VDAC1 mutant (bottom panel). Recombinant wt-VDAC1 or S215E mutant was reconstituted into planar lipid bilayers. Representative current recordings with the corresponding amplitude histograms are shown. Current was monitored before and after addition of HKII in response to a −30 mV test potential during a 30-second duration. Downward deflections denote channel opening. The dashed blue lines denote 0-current levels.

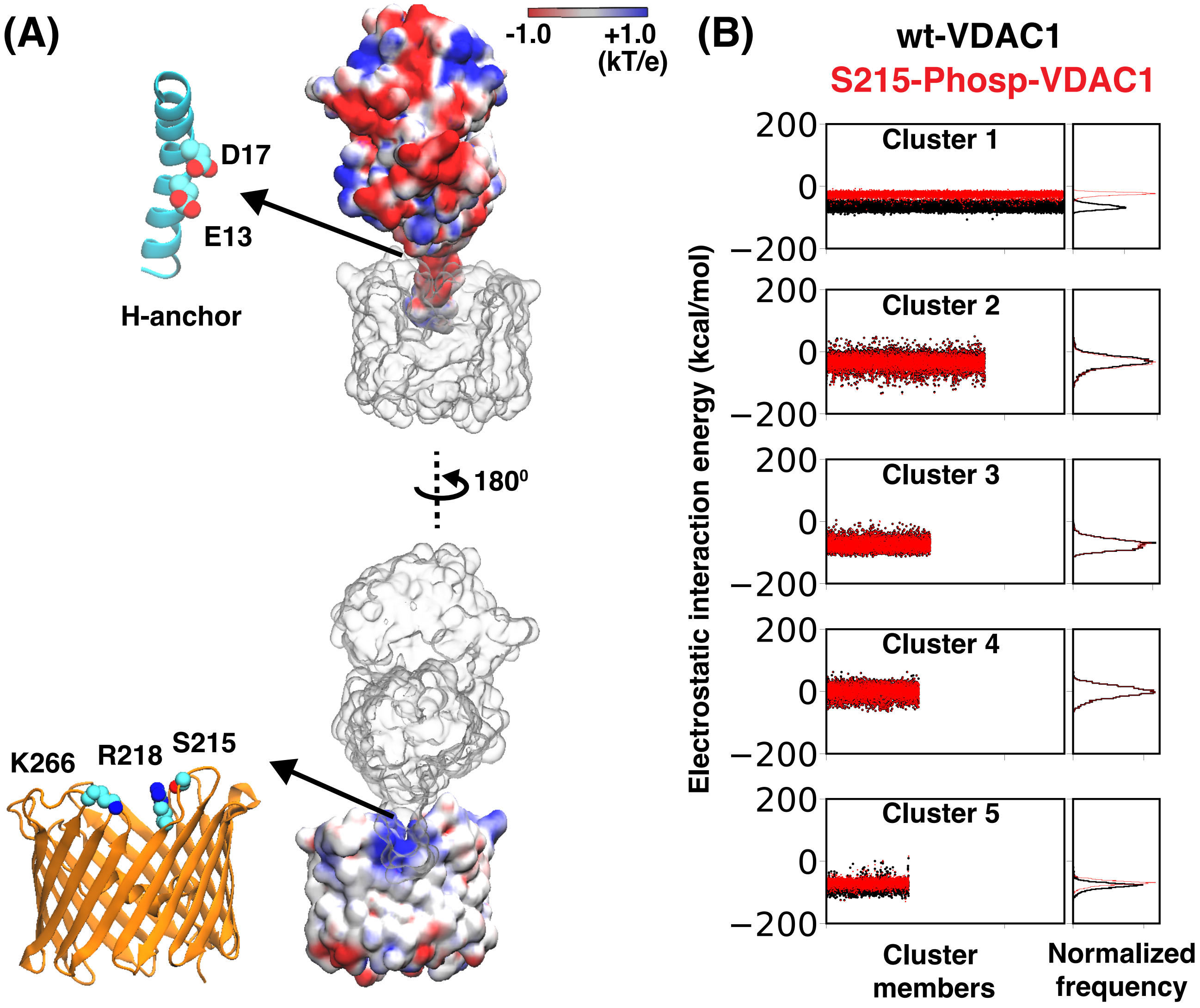

Figure S15. Proposed disruption mechanism of HKII/VDAC1 complex. (A) Electrostatic potential map of HKII-N and VDAC1 in HKV1 (generated using the Poisson-Boltzmann (PB) equation solver module in CHARMM-GUI[5–7]), highlighting the binding interface near H-anchor. The interface contains a electronegative surface in H-anchor and a electropositive surface in VDAC1. (B) Electrostatic interaction energy calculated between HKII and VDAC1 in two different states of VDAC1: wt-VDAC1 and S215-Phosp-VDAC1. The calculations were performed for all members of the five clusters (cluster 1 to 5) derived from BD simulation of wt-VDAC1.

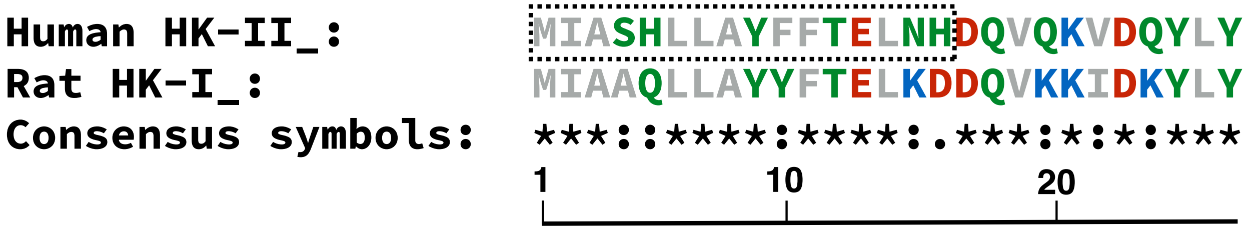

Figure S16. Sequence alignment of H-anchor of human HKII and rat HK-I. Residues from 1 to 16 of human HKII (highlight with dotted box) is missing from the crystal structure (PDB ID: 2NZT). Description of the consensus symbols: An * (asterisk) indicates positions that have a fully conserved residue, A : (colon) indicates conservation between residues of strongly similar properties, A . (period) indicates conservation between residues of weakly similar properties. Each residue is represented by their one-letter amino acid abbreviation and colored based on their residue type: Gray representing hydrophobic, green polar, red acidic, and blue basic residues. The alignment was performed on full-length human HKII and full-length rat HK-I using the Clustal Omega[8] alignment tool within Uniprot[9]. Full-length human HKII and rat HK-I share an overall 73% sequence identity.

References

1. Bergdoll, L. A. *et al.* Protonation state of glutamate 73 regulates the formation of a specific dimeric association of mVDAC1. *Proc. Natl. Acad. Sci.* **115**, E172–E179 (2018).
2. Abu-Hamad, S., Zaid, H., Israelson, A., Nahon, E. & Shoshan-Barmatz, V. Hexokinase-I protection against apoptotic cell death is mediated via interaction with the voltage-dependent anion channel-1: mapping the site of binding. *J. Biol. Chem.* **283**, 13482–13490 (2008).
3. Pastorino, J. G., Hoek, J. B. & Shulga, N. Activation of glycogen synthase kinase 3β disrupts the binding of hexokinase II to mitochondria by phosphorylating voltage-dependent anion channel and potentiates chemotherapy-induced cytotoxicity. *Cancer Res.* **65**, 10545–10554 (2005).
4. Smart, O. S., Neduvelil, J. G., Wang, X., Wallace, B. & Sansom, M. S. HOLE: a program for the analysis of the pore dimensions of ion channel structural models. *J. Mol. Graph.* **14**, 354–360 (1996).
5. Jo, S., Kim, T., Iyer, V. G. & Im, W. CHARMM-GUI: a web-based graphical user interface for charmm. *J. Comput. Chem.* **29**, 1859–1865 (2008).
6. Im, W., Beglov, D. & Roux, B. Continuum solvation model: computation of electrostatic forces from numerical solutions to the poisson-boltzmann equation. *Comput. Phys. Commun.* **111**, 59–75 (1998).
7. Jo, S., Vargyas, M., Vasko-Szedlar, J., Roux, B. & Im, W. PBEQ-Solver for online visualization of electrostatic potential of biomolecules. *Nucleic Acids Res.* **36**, W270–W275 (2008).
8. Sievers, F. *et al.* Fast, scalable generation of high-quality protein multiple sequence alignments using Clustal Omega. *Mol. Syst. Biol.* **7**, 539 (2011).
9. The UniProt Consortium. UniProt: a worldwide hub of protein knowledge. *Nucleic Acids Res.* **47**, D506–D515 (2019).
