# Supplementary material for: Structural basis of complex formation between mitochondrial anion channel VDAC1 and Hexokinase-II
Source: Commun Biol. 2021 Jun 3;4:667. doi: 10.1038/s42003-021-02205-y (PMC8175357; doi:10.1038/s42003-021-02205-y)
Supplement: Supplementary file 2 — Description of Additional Supplementary Files [file 42003_2021_2205_MOESM2_ESM.pdf]

Description of Additional Supplementary Files

File name: Supplementary Movie 1

Description: A representative BD trajectory capturing the formation of the HKII/VDAC1 complex

File name: Supplementary Movie 2

Description: MD trajectory of HKV1 (Run1)

File name: Supplementary Movie 3

Description: MD trajectory of HKV1 (Run2)

File name: Supplementary Movie 4

Description: MD trajectory of HKV2 (Run1)

File name: Supplementary Movie 5

Description: MD trajectory of HKV2 (Run2)

File name: Supplementary Movie 6

Description: MD trajectory of HKV3 (Run1)

File name: Supplementary Movie 7

Description: MD trajectory of HKV3 (Run2)

File name: Supplementary Movie 8

Description: MD trajectory of HKV4 (Run1)

File name: Supplementary Movie 9

Description: MD trajectory of HKV4 (Run2)

File name: Supplementary Movie 10

Description: MD trajectory of HKV5 (Run1)

File name: Supplementary Movie 11

Description: MD trajectory of HKV5 (Run2)
